# Remote Patient Activity Monitoring System by Integrating IoT Sensors and Artificial Intelligence Techniques

**DOI:** 10.3390/s23135869

**Published:** 2023-06-25

**Authors:** Preethi Palanisamy, Amudhavalli Padmanabhan, Asokan Ramasamy, Sakthivel Subramaniam

**Affiliations:** 1 Department of Computer Science and Engineering, Kongunadu College of Engineering and Technology, Trichy 621215, India; 2 Department of Computer Applications, B. S. Abdur Rahman Crescent Institute of Science and Technology, Vandalur 600048, India; amudhavalli@crescent.education; 3 Department of Electronics and Communication Engineering, Kongunadu College of Engineering and Technology, Trichy 621215, India; asokece@gmail.com; 4 Department of Computer Science and Engineering, Sona College of Technology, Salem 636005, India; sakvel75@gmail.com

**Keywords:** monitoring, COVID, Application Peripheral Interface, sensors, inception, deep learning, CNN-UUGRU

## Abstract

Even with the most cutting-edge tools, treating and monitoring patients—including children, elders, and suspected COVID-19 patients—remains a challenging activity. This study aimed to track multiple COVID-19-related vital indicators using a wearable monitoring device with an Internet of Things (IOT) focus. Additionally, the technology automatically alerts the appropriate medical authorities about any breaches of confinement for potentially contagious patients by tracking patients’ real-time GPS data. The wearable sensor is connected to a network edge in the Internet of Things cloud, where data are processed and analyzed to ascertain the state of body function. The proposed system is built with three tiers of functionalities: a cloud layer using an Application Peripheral Interface (API) for mobile devices, a layer of wearable IOT sensors, and a layer of Android web for mobile devices. Each layer performs a certain purpose. Data from the IoT perception layer are initially collected in order to identify the ailments. The following layer is used to store the information in the cloud database for preventative actions, notifications, and quick reactions. The Android mobile application layer notifies and alerts the families of the potentially impacted patients. In order to recognize human activities, this work suggests a novel integrated deep neural network model called CNN-UUGRU which mixes convolutional and updated gated recurrent subunits. The efficiency of this model, which was successfully evaluated on the Kaggle dataset, is significantly higher than that of other cutting-edge deep neural models and it surpassed existing products in local and public datasets, achieving accuracy of 97.7%, precision of 96.8%, and an F-measure of 97.75%.

## 1. Introduction

As the world progresses towards real-time and remote monitoring, along with rapid disease diagnosis, remote healthcare has emerged as a significant area of research. Remote healthcare encompasses a range of practices that utilize technology to monitor patients outside of traditional hospital settings. This includes approaches such as mobile health and telehealth. The advantages of remote patient monitoring include the capability to fully monitor sick people, the prevention of diseases that harm them and early deaths, a lowering in the number of hospital admissions, cost savings from hospital admissions, efficiency gains in healthcare services, and the capability to obtain additional correct information outcomes while allowing patients to maintain their normal activity. Those that gain from remote patient monitoring include those with chronic illnesses, patients recovering from surgery, people with mobility issues or additional disabilities, senior patients, and babies. All of these people have medical conditions that require ongoing care. Making every patient’s daily life as enjoyable as possible is the aim of outstanding healthcare [1].

A more advanced subfield of artificial intelligence (AI) is machine learning (ML). Smarter machines can be built using artificial intelligence. ML is a strategy for implicitly changing learning from experiences and samples. Instead of writing code, data are added to a general algorithm, and analysis is built based on the provided information. Online searches, banner advertising, spam detection, and trading platforms are just a few of the applications that use machine learning [2]. By analyzing vast volumes of data and automating the labor of information scientists, machine learning has gained the same attention and significance as cloud computing and big data. Numerous fields, interpersonal organizations, and businesses, including those in design sciences, biogenetic research, and security, collect and analyze formative information on an extensive scale. Most traditional ML techniques are created for information that is fully stored within the structure [3].

Patients who have been involved in an accident or have suffered serious injuries might only be electronically monitored throughout their ambulance ride to the hospital. However, the focus is on ensuring a secure transition to remote monitoring, and the health center supports the delivery of immediate medical care in the most life-threatening circumstances. Doctors can monitor a patient’s progress or preservation while also providing any necessary advice to paramedics who are physically there with the patient. A data-gathering system, a healthcare end terminal, an information-processing system, and a communication network are the four essential components of a remote-monitoring system. Devices with sensing capabilities and multiple sensors that may wirelessly send information comprise an information-gathering system [4]. Since technology has advanced, sensors may now include cameras and mobile phones in addition to medical sensors. Recent studies on frictionless medical techniques, in which medical tools do not directly contact individuals, are the basis for this technology. The most common type of sensor is used in noncontact approaches in wireless sensor networks (WSNs). Personal area networks (PANs), wireless body area networks (WBANs), and body area networks (BANs) could be used to further categorize them. A system for processing information comprises a processing unit or circuitry, as well as a system that may receive and send information. A database as a specialized gadget, or even the physician’s smartphone, might serve as a medical device. The data processing system and the information collection system are connected by the core communication network, which also sends the findings and conclusions to a healthcare practitioner who is connected to the system.

The definitions and general design for remote monitoring of patient systems are introduced in the following section, which is then followed by the benefits of RPM. The crucial RPM components are then discussed. The study is then completed in Section 4 after the data are statistically analyzed. The major contributions of the proposed taxonomy are as follows:

- This study focuses on artificial-intelligence-based IoT technology. The system was built with three tiers: a cloud layer using an Application Peripheral Interface (API) for mobile devices, a layer of wearable IoT sensors, and an Android mobile application layer, where the IoT perception layer collects data from the wearable sensors with advanced MMS systems and incorporates ZigBee for better computational handling.
- The CNN-UUGRU model combines convolutional and updated gated recurrent subunits to achieve high accuracy in activity recognition.
- The sensed data can be utilized to issue a warning to nearby residents, advising them to exercise extreme caution and take preventive actions. The technology has already been used to link physiological measurements to routine tasks.
- The remaining sections of the paper are organized as follows: In Section 2, previous studies relevant to our research are discussed. Section 3 introduces the fundamental concepts of the techniques employed in the proposed system, including the Tier-Based Working Model and the Data Prioritization task using the Deep_Convolutional-LSTM. Section 4 provides a comprehensive analysis of the proposed scenarios and presents a comparison between our study and previous works. Finally, Section 5 presents the conclusion of the study and provides recommendations for future research directions.

## 2. Literature Review

The framework of the existing literature is as follows: First, we describe human behavior and the various HAR approaches, along with the advantages and disadvantages of relying on an embedded system. Following a thorough discussion of the online vs. offline approach and the numerous machine learning approaches used in earlier research for sensor-based human-action recognition, the advantages of hybrid neural network designs over other traditional methods are discussed.

### 2.1. Human Activity

Movements, hand gestures, and other physical actions that entail the release of energy are referred to as human activity [5]. Examples include actions such as eating, drinking, and walking. Human actions are simple or complicated. Simple human activities take into account body motion and posture to define the different activities. Jogging, walking, and running are examples of these activities. Human behaviors are composed of fundamental acts and a function [6]. Imagine, for instance, a task where the individual just sits and executes the action of eating. Simple and complex human behaviors will be categorized into three groups using deep learning techniques dependent on smartphone and wearable sensor readings.

### 2.2. The Human Activity of Recognition Methods

**Figure 1:**
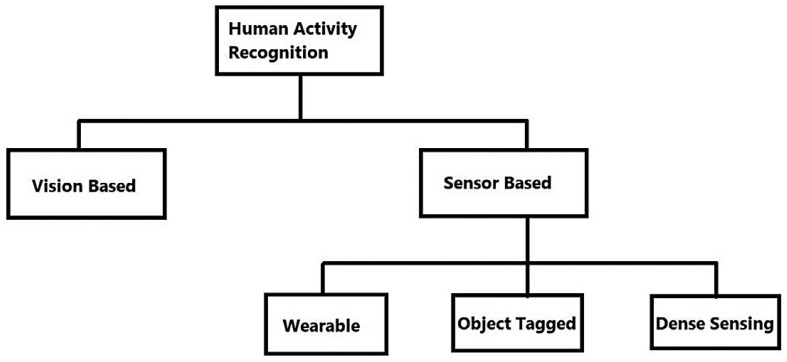
Recognition of human activity in various aspects.

Human activity recognition systems may be divided into two categories: vision-based and sensor-based systems [7], as shown in Figure 1. Three other kinds of sensor-based technology may be identified: (a) clothing; (b) device-free; and (c) device-bound (object-tagged) (dense sensing) [8]. A visual sensor-based system employs cameras and movies to record and catalog the behaviors of the research participants. Privacy is the key worry, since it may not be viable to put cameras at all sites owing to compliance and regulatory restrictions. Additionally, computer power is required to analyze photos and videos using computer vision-based approaches. One sort of sensor modality that may be used to recognize and locate human behaviors is wearable sensor technology. These sensors may be embedded in bands, smart watches, clothing, and smartphones, or worn on the body. Numerous IoT-based sensor devices that may be worn on the body to record movements, including accelerometers, gyroscopes, magnetometers, electromyographs (EMGs), and electrocardiographs (ECGs), are readily accessible on the market [9]. The appropriate positioning of wearable sensors directly affects the activity data being gathered from a person. The most obvious places to put the sensors are the breastbone, lower back, and waist. The exemplification of body motions improves with sensor placement relative to the center of mass. This is where the use of smartphones and smartwatches that have accelerometers and gyroscopes is highly practical. Smartphones are placed in pockets, whereas wearables and watches are worn on the dominant arm. To recognize complicated hand-based human behaviors, such as teeth brushing or sipping soup, both technologies may be very beneficial. In sensor-bound or entity systems, detectors are affixed to commonplace items. Individuals’ interactions with these objects are used to identify certain activities. The user is compelled to use the item to which the gadget is linked, which is comparable to the drawback of wearing sensors. Such gadgets include microphones and IR sensors. Device-free detectors, also known as environment-based or dense different sensors, offer the advantage of not requiring the person to wear anything on their body. These sorts of non-device detectors may be directly placed in the surroundings and can capture the subjects’ movements, interactions, and action-based tasks. The most popular detectors are RFID, FM and microwave, Wi-Fi, and ZigBee Radio [10], which are common radio-frequency-based detection types.

## 3. Proposed Methodology: Tier-Based Working Model

**Figure 2:**
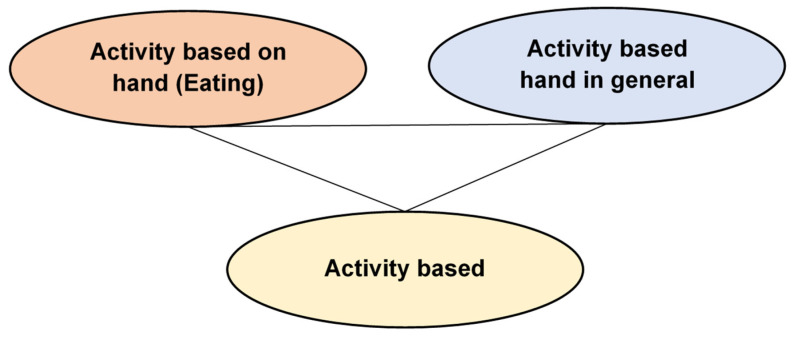
Activities in the Wireless Sensor Data Mining (WSDM) database.

This section explains the wearable sensors for remote healthcare monitoring systems. The physical features of physiological signals are depicted in Table 1. Figure 2 shows the three-phase system.

The system is made up of:

(1) WBAN, or wireless body area network
(2) IPDA-based personal server (PPS);
(3) A Medical Server for Healthcare Monitors (MSHM).

Phase 1

The patient is the system’s user. Wearable technologies establish a WBAN to detect the vital signs of patients and deliver feedback to maintain proper health [11]. Medical sensors include five essential parts:

(1) Sensor: A sensor chip used to gather physiological signals from the patient’s body.
(2) Microcontrollers: This device is utilized for local data acquisition, such as compression algorithms, and it also regulates the operation of other detector network devices.
(3) Memory: This is used to temporarily store the detected information.
(4) Radio transceivers: Send/receive wireless physical information between nodes.
(5) Power source: The battery-operated sensor nodes have a lifespan of many months [12].

Detectors can detect, collect, and analyze one or more physiological parameters. An EKG sensor may be used to monitor cardiac activity, a blood flow meter can be used to regulate blood pressure, a respiration detector can be used to detect respiration, an EMG device can be used to monitor muscle contraction, and an EEG device can be used to detect neurological activity.

**Figure 3:**
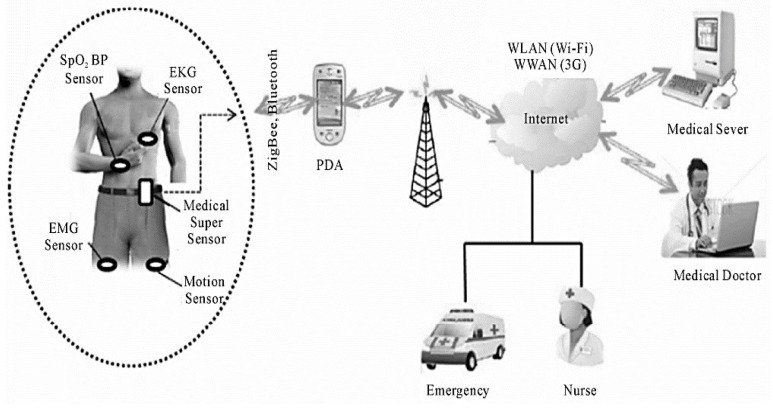
Design of remote medical surveillance systems using wearable sensors.

We designed a WBAN that incorporates a high-tech sensor, the MSS. Compared to other networks, this detector has more storage, processing power, and connections (Figure 3) to communicate with body sensors through the MSS and the personal server via ZigBee.

Both the Bluetooth and ZigBee protocols were considered in this concept. The Wireless specification only permits a maximum of seven functionality slaves. This device will contain more than seven sensing devices; thus, Bluetooth is not an option.

Second, the ZigBee/IEEE 802.15.4 standard is equipped with a low-cost, low-power, short-range architecture that can manage huge sensing devices with up to 65,000 nodes and provide reliable information transport. It utilizes the Industrial, Scientific, and Medical (ISM) free band, or 2.4 GHz, and can handle up to 250 kbps. ZigBee transmits physiological data from WBAN to the patient network.

The following list includes further justifications for its usage:

Security: Protecting patient information is essential; no unauthorized person should modify it. There is a requirement for the secure transmission of data from the WBAN to individual and medical servers. The aggregate data transferred between an MSS and a personal server via ZigBee are encrypted using AES 128.
Scalability: It is very scalable over a wide range of devices. Regardless of the manufacturer, there should be interoperability between numerous medical and non-medical devices and information management devices. The MSS undertakes inconspicuous sampling, gathers vital signs via sensing applications, checks out unnecessary data, reduces the vast number of details given by BSNs and saves them quickly, and analyzes and sends essential patient data using wireless ZigBee/IEEE 802.15.4. This increases overall bandwidth usage and decreases BS energy consumption since networks are not required to send information to the IPDA but to the MSS, which is closer to the BSs, increasing the battery capacity of each sensor network.

Phase 2

Individual Server

The personal server interfaces with WBAN nodes using ZigBee. It is used with smart IPDAs. It retains patient IDs and uses the hospital’s Internet address to communicate with hospital assets. It collects physiologically essential signals from the WBAN, evaluates them, and prioritizes the transfer of critical information when there is a major clinical alteration in the current patient condition or entire database, such as changes in circulation impulses, temperatures, or oxygen saturation, and then sends it to the regional hospital.

Furthermore, the IPDA is capable of automatically evaluating physiological data and performing local reasoning to assess the user’s health state based on information obtained from the MSS, and giving feedback through a user-friendly and engaging GUI.

Phase 3

Servers and personal servers are connected through 3G communications, although other longer-range communications, including GPRS and WWANs, may also be utilized.

The IPDA has two distinctive services that enhance transmitting data latency, bandwidth, and energy consumption. They are information minimization and priority sequencing.

**Table 1:** Physical features of physiological signals.

Signal of Physiological	Range of Factor	Data Rates (Kbps)	Arriving Time (s)
Electrocardiograph (EKG)	0.5–4 mw	6.1	0.003
Body temperature	32–40 degree	0.0025	5
Respiration rate	2–50 breaths/min	0.25	0.06
Never potentials	0.02–3 mv	241	5 × 10^−5^
Blood pressure	10–400 mmHg	1.3	0.02
PH value of blood	6.9–7.9 PH units	0.049	0.26
Flow of blood	1–300 mL/s	0.49	0.026
Oxygen saturation (SpO_2_)	0.02–0.86/s	2.4	0.17

### 3.1. Data Compression and Prioritization of Tasks

Different physiological data are sent between sensor devices and patient servers. Transmissions are categorized by data rate and delay. They fit into the following categories:

⮚ Higher rate of data and lower traffic latency;
⮚ Lower rate of data and lower traffic latency;
⮚ Lower rate of data and higher traffic latency;
⮚ Higher rate of data and higher traffic latency.

Low latency indicates that the duration it takes for a system to respond to the transmission of a crucial signal should be as short as feasible, while a high data rate means that the signal must be sent quickly and reliably.

Each physiological indicator has priority weight according to Table 2 below. It shows the order in which physiological signals will be conveyed from the IPDAs to the hospital through 3G connectivity for additional assessment and care by medical personnel.

Priority 1 physiological signs have the highest importance and will be communicated first. The vital indicator should be given right away. It indicates that the patient needs urgent medical attention, although higher data rates and lower latency signals are unimportant. It will be compressed to a specific proportion and stored in the IPDA’s local memory for later communication when higher-priority physiologic indicators, including priority 1 and priority 2, have already been broadcast.

As previously mentioned, priority access to a patient’s health records is essential to ensure appropriate care and to increase safety and quality of care. Priority scheduling lowers transmission delays for physiological signals and transportation congestion. By using data compression, the overall amount of data delivered is decreased. Because of the increased bandwidth use, the overall transmission time is decreased. However, the IPDA relies on a battery for power and uses a lot of energy when transmitting [13].

This method saves energy on the IPDA because it only sends the most important vital signs first. The less important signs are saved and sent later. The flowchart illustrating the IPDA functioning modes may be seen in Figure 3 below. Active Mode and Inactive Mode are the available modes.

To conserve energy, the IPDA is idle when it has no information to acquire from the MSS or communicate to the patient database, but it wakes up promptly to receive information and keep it. It prioritizes all acquired physiological data and relays them to the healthcare server so healthcare professionals may be suitably prepared before such a patient is brought in or dispatched to save his/her life.

### 3.2. Medical Server for Healthcare Monitors (MSHMs)

Phase 3 is referred to as the MSHM. It accepts information from a personal server and supports the design. It is located in hospitals that provide healthcare services. It is clever because it can learn patient-specific thresholds and gains knowledge from a patient’s prior medical data [14]. The MSHM maintains EMRs for all patients who have registered, which are accessed online by various medical staff members, including medical doctors, specialists, and physicians inside the hospital. The doctors know the patient’s situation. The MSHM’s duties are maintaining the user identity, getting information from personal servers, preparing and entering the information into EMRs, and assessing statistics. The patient’s physician may monitor information and trends from his or her office through the internal network or Internet to ensure that health metrics are fulfilled. If information is outside of its limits or reveals significant health issues, emergency unit personnel may be notified. If the patient is located in a distant place, a specialist physician will assess the patient’s physiological data, make a diagnosis, and recommend the appropriate course of action and medications. This information will be transmitted online to the remote doctor. Additionally, the MSHM gives patients feedback suggestions, such as workouts recommended by doctors.

### 3.3. Implementation and Evaluation

The findings were generated using Tensorflow 2.4.0, Keras 2.4.3, Python 2.6.9, Pandas 1.1.5, a Numpying raw gyroscope, and acceleration location information from WISDM, and the parameters are given in Table 3 for information.

#### 3.3.1. Keras Model Setup

**Figure 4:**
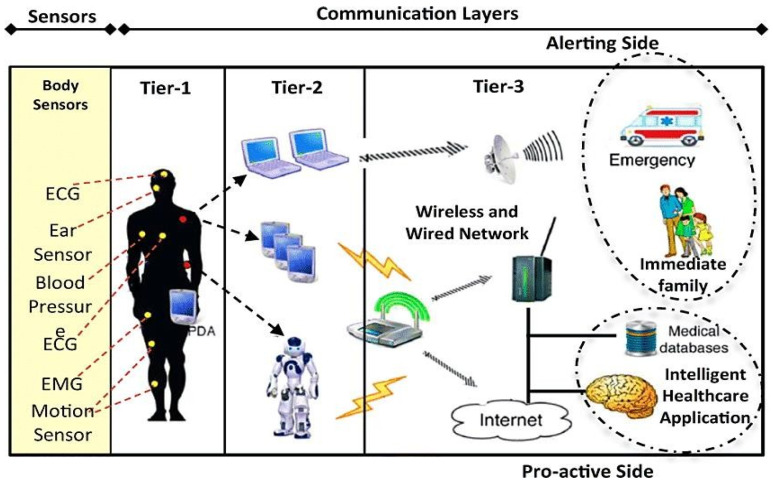
Tier-wise workflow diagram.

This method’s setup includes several DL models which are covered in depth in the following subsections [15]. The CNN-UUGRU hybrid model architecture shown in Figure 4 is employed for wristwatch and smartphone datasets. It can scan huge star patterns as bricks, extract features, and then use the UGRU layer to build the functionalities. Because a standard picture consisting of 10 s, or 200 time steps, was used, the time stages included in each sequence were broken down into four subsets consisting of 50 time stages each before being input into the CNN together with six elements. To enable the CNN architecture to read all four input subgroups, a Time Distribution layer is wrapped around it. A max-pooling level, with dropouts to reduce the false positive rate, and which flattens and feeds into two layers of stacked UGRU dropouts, and SoftMax for classifiers, are used to depict a two-layer 1D CNN. Given that classification is being performed on the information, Cross-Entropy Losses are used as the linear transformation. Cross-Entropy Losses for multi-class segmentation are defined as:

(1) $$J=1/N\left(\sum_{i=1}^{N}(Y_{i}-log\;({\hat{y}}_{i}\right)))$$

where *Y_i_* is the true label, ${\hat{y}}_{i}$ is the label to be precisely cited for the *i*th learning sample, and *N* is the overall training data.

OptKeras is a package manager for Bayesian hyper-parameter optimization utilizing the Optuna module. Optuna automates the deep convolutional neural network and machine intelligence hyper-parameter tuning using Bayesian optimization. Table 4 shows the CNNs-UGRU hyper-parameters configured utilizing OptKeras.

#### 3.3.2. Inception Time

The Inception Time deep learning model setup uses McFlyAutoML. This package’s benefit is that it makes it simple to generate numerous models, then allows one to choose the best model based on an assessment measure after utilizing a random search to refine the models. In this research, four cutting-edge designs were constructed using McFly, and the best was picked. This design contains five activation functions, each having a max-pooling layer and four Conv 1D layers, three with varying kernel sizes and one with a constant kernel size. The time information from the smartwatches and smartphones dataset, which is processed using a dynamic panel model of 10 s, is the input to this model. The last layer concatenates conception modules’ input and averages it globally to decrease parameters [16]. The hyper-parameters for the wearable device databases are chosen randomly, as shown in Table 4.

**Table 4:** Hyper-parameters for Inception Time.

Factors	Ranges
No. of filters	73
Rate of learning	0.0278
Size of maximum kernel	34
Depth of network	6
Rate of regularization	0.0198

#### 3.3.3. Deep_Convolutional-LSTM

Deep_Convolutional-LSTM is a deep learning hybrid model. It comprises multiple Conv2D layers, followed by an LSTM layer. It was developed in the study using McFlyAutoML. The advantage of using McFly is that it is very straightforward to construct an ML model by randomly selecting significant energy for numerous models. In this study, four Deep_Convolutional-LSTM techniques were constructed, and the best one was selected depending on recognition rate. The prototype has seven Conv2D layers using various filter lengths to choose local features from input time series analysis, followed by three stacked LSTM layers. The quantity of layers is a parameter that is controlled. To categorize various human behaviors after the LSTM is introduced to reduce overfitting, a TDL and max-pooling are used. The smartwatch dataset hyper-parameters are picked randomly (Table 5).

## 4. Numerical Results and Deliberations

The suggested solution uses a web-based app for medical experts and an Android app for the patient or family responders. The two interfaces are synced to gather and notify healthcare information. To learn more about the three-part proposed system of IoT-based smartwatches, further details are provided. PIPs wear embedded sensors on their wrists or ankles, or wristbands to identify physiological health concerns and transmit the position and localization to the API cloud management software. Two or more people interact at the receiver portion. The user login’s close relative receives a caution and alerts about the patient’s health. The API is hosted on a webpage with cloud-style information storage and retrieval. The serious issue involving the provision of drugs and counseling sessions is monitored and managed by medical experts using this API. The Android application created for smartphones is synced with the API interface to provide regular alerts of the patient’s health problems while they are under confinement.

### 4.1. Device Evaluation Outcomes

The gadget was attached to the human body and went through several tests while being worn by the user, and also recorded information on heart rate, temperature, location, SpO_2_, and coughing. A source of heat from outside was utilized to assess how well heat measurement works in the temperature settings. Due to COVID-19, the patient’s limitations were exposed and implemented. The situation examined is described in the sections that follow. With a successful completion time of 1 h and a synchronization time of 10 min, the device’s operational time was determined.

### 4.2. Observation of Someone Displaying Normal Symptoms

**Figure 5:**
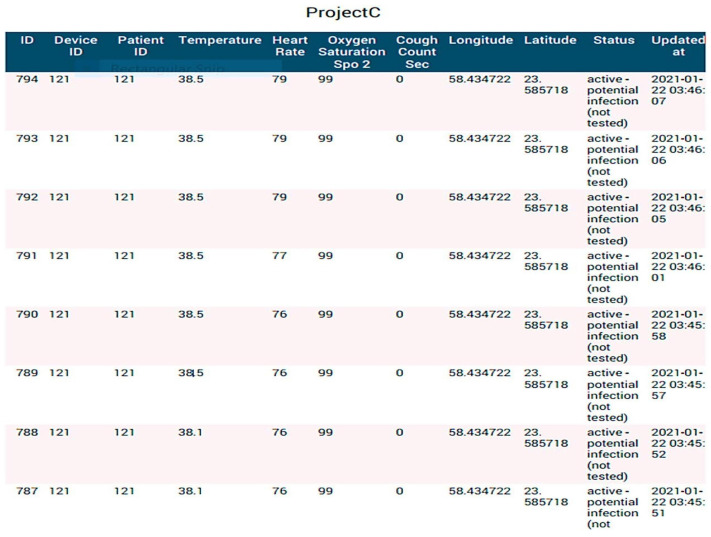
Sample system report showing potential infection.

The practice management portal tracks and uploads temperature, SpO_2_, and pulse data into the cloud. Moreover, the gadget provides periodic position data, comprising the longitude and latitude of the webpage. An Arduino BLE sensor 33 is used to analyze coughing noises every 2 s and sends the findings to a webpage for storage and processing. The person’s location will be shown by a green indicator on the dashboard map. The technique underwent testing at ambient temperature. Figure 5 illustrates a person with regular indicators, suggesting that the individual has no probable COVID-19 signs, where the temperature is within the average limits (T 37), the oxygen level content is within standard parameters (SpO_2_ > 94), the heart rate is at a reasonable level (120), and the person does not exhibit cough waveforms. During the enrolment phase, a patient ID and device ID are issued. The patient ID and the vehicle’s license plate are presented here. Longitude and latitude represent localization, which will not be seen until the gadget submits the information to the webpage. All sensor information is sent to a cloud-based system, which analyzes the measurements and decides on the final state—in this instance, actively safe—by comparing the current numbers with the allowable ranges.

### 4.3. Tracking of Individuals Displaying Potential Infection Symptoms

**Figure 6:**
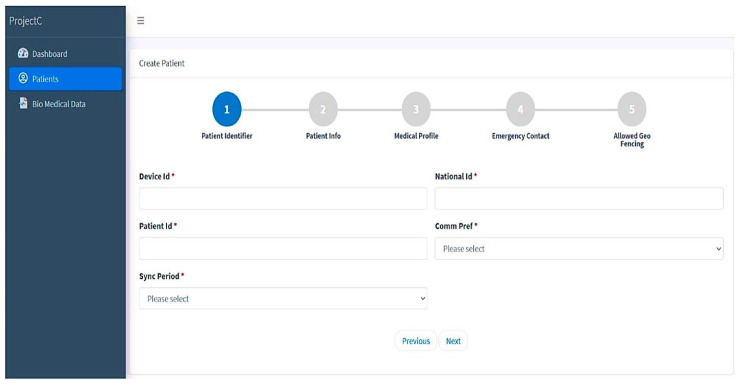
New patient registration (* indicates that the fields are mandatory).

**Figure 7:**
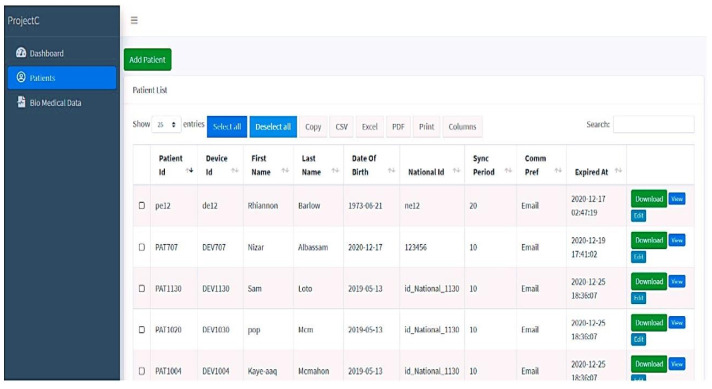
Details of registered patient.

The temperature is the primary COVID-19 indicator according to the WHO [17,18,19,20], and anybody with a thermometer reading of 37.5 °C or above may be infected. A report generated by the system—which was operating at high temperatures—is shown in Figure 6 and Figure 7. It is also possible to monitor other indicators including coughing, SpO_2_, and pulse. It is important to understand that the cough count is determined by adding up all of the cough waveforms, which helps the case manager determine the level of the symptoms and avoids any false positives. If (Temperature > 38; SpO_2_ > 94; Cough frequency > 10), the system will indicate (active probable disease, not verified). More importantly, the technique indicates a red color on the map to let the guidance counselor know that the person who was tested could start showing signs of a possible COVID-19 disease. The job will be on the system dashboards that display the number of impacted users (Figure 5).

### 4.4. Notifying in Case of Self-Quarantine Breach

The wearable device is supported by GPS Shielding, which provides periodic updates on the patient’s latitude and longitude measurements. The patient must provide the initial location, which must then be uploaded to the device to set the initial position. The PIP shows up on the map with his or her location after the location has been submitted. A distance of 5 km of virtual movement was used to test device performance. The device sends a notification that the patient has left the quarantine zone as soon as the PIP leaves the area. This design’s advantage is that it provides a mild reminder that the individual has left the quarantine area, and a notification that they have done so should alert residents to such a circumstance. This can assist in deployment and maintaining the PIP by often indicating their location.

### 4.5. Case Manager for Patients

The probably infected person (PIP) should be registered on this website by selecting new patients as the initial step. Five consecutive stages are required for this registration, which outlines all the conditions for patients who are under quarantine (see Figure 6). In the initial phase, patients are registered, providing the relevant data including name, age, and national ID. The medical history and specifics of the probably infected patient are covered in the next stage. Further stages include emergency contact and initial geographical location or planned quarantine place to follow the patient’s condition. Case managers have access to their patient’s records at all times and may use labels to signify the patient’s current health state. The patient’s health status is shown on this screen along with their patient ID and device ID. For monitoring, analysis, and documentation, all pertinent information is kept and preserved. Following registration, the patient information is retrieved in various text forms from the database that has been saved.

### 4.6. The Website with Biomedical Data

**Figure 8:**
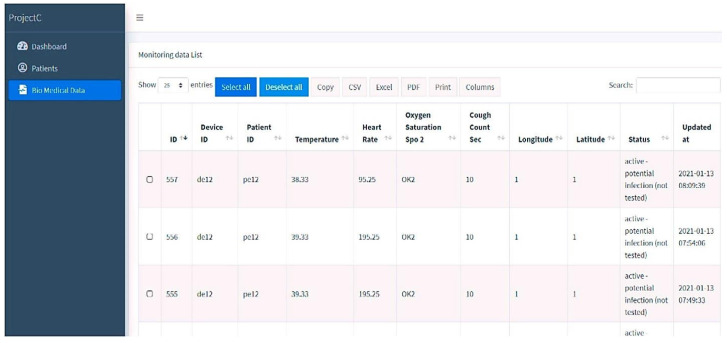
Biomedical data of the registered patient.

The hardware devices on the website are used to collect, upgrade, and synchronize time by modifying the worn sensor’s information. The device continuously monitors the patient’s pulse, core temperature, SpO_2_, and coughing. This interface also must use a GPS sensor to track the patient’s position while under quarantine. In Figure 8, the patient’s position, including the most recent latitude and longitude, is updated. Since the privacy of the data in this module is crucial, as it relates to an individual’s health status, adequate measures should be taken to ensure secure data collection, transmission, and storage to protect against unauthorized access or breaches. In addition to biomedical data, the website also utilizes a GPS sensor to track the patient’s position during quarantine. This raises concerns regarding the collection and storage of Personally Identifiable Information (PII), such as the patient’s location, which can potentially identify the individual. Safeguards should be implemented to protect the confidentiality of PII and limit access to this information to authorized personnel only. Thereby, the website can enhance the protection of individuals’ sensitive biomedical and location data, fostering trust and ensuring compliance with privacy regulations.

### 4.7. Device Dashboard

**Figure 9:**
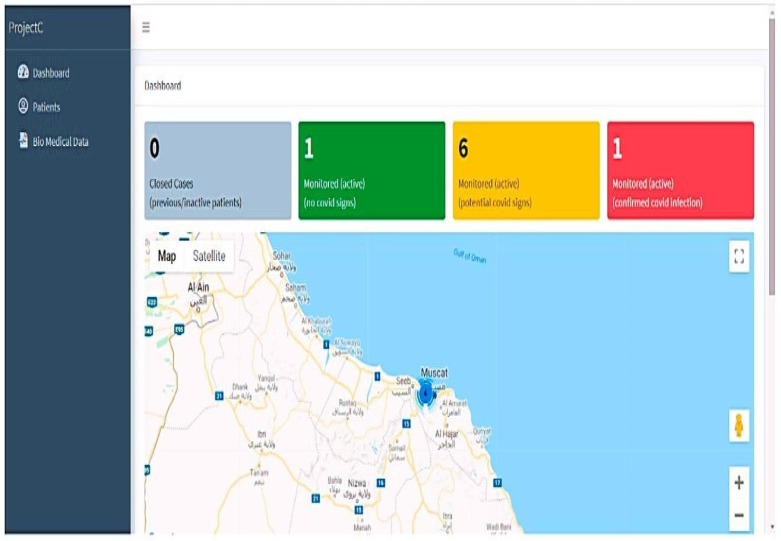
Dashboard with COVID-19 status indicators in different colors.

The dashboard displays the status graphically by monitoring those with no COVID-19 signs, probable indicators, and verified COVID indications, and constantly refreshes the data from the linked devices every minute. Normal physiological signals are indicated by the color green, people with chronic health concerns by the color orange, verified instances by the color red, and concluded instances by the color black. The system’s colored counter provides a comprehensive picture of the patient count state. The main two statuses are probable infections and cases that have been positively identified as having infections. The dashboard tracks a person’s position and synchronizes these data with the biological data in a sheet. The display also summarizes the overall number of confirmed instances and closed cases. The patient’s transgressions and the pertinent geofencing subcategories are shown in Figure 9 below.

**Figure 10:**
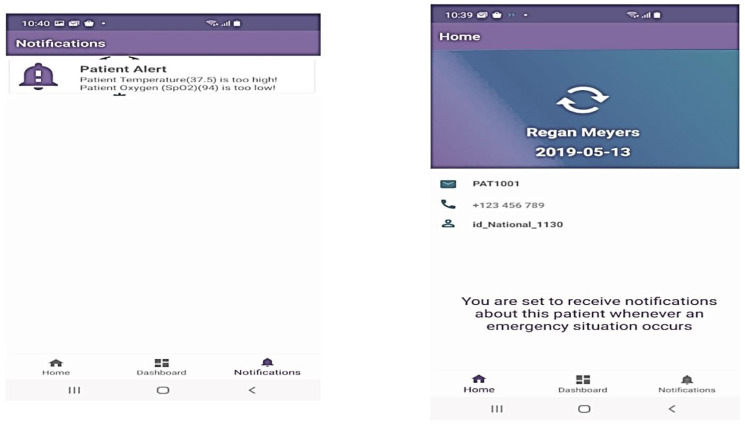
Android app dashboard.

The Android app notifies possible infected patients and their families about their health, acting as a home control to communicate with the doctor on their behalf. The Android-based application’s interface consists only of the home, dashboard, and notification portions. First, the patient’s family responder’s cellphone number is registered with the patient ID. After identification, the responder may receive alerts based on human physiological vital signs in the announcements area. The following paragraphs outline the main aspects of the Android-based app:

- Successfully registers a smartphone with a patient ID to receive their status.
- Keeps a patient’s health records in chronological order and contacts emergency services. Figure 10 displays the android app page with his patient ID and the real-time notification dashboards.

### 4.8. Wearable Sensing Devices of 3D Views

The 3D mobile-sensing gadget-level information is gathered using heart rate sensors, temperature monitors, a spark fun pulse analyzer, an audio detector, an Arduino MKR 1400 motherboard GSM, an Arduino Nano 33 BLE sensor, and an external lithium polymer battery. All elements are 3D waterproof devices that encrypt web API communication. The three-dimensional design has a moveable form with a one-piece housing and a microcontroller, sensors, GPS, and a power source.

**Figure 11:**
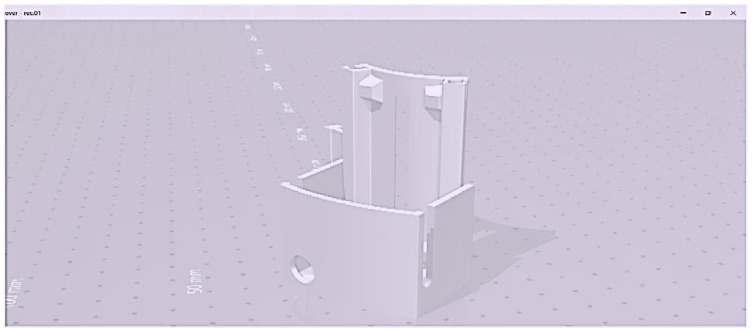
Three-dimensional model for SketchUp software.

**Figure 12:**
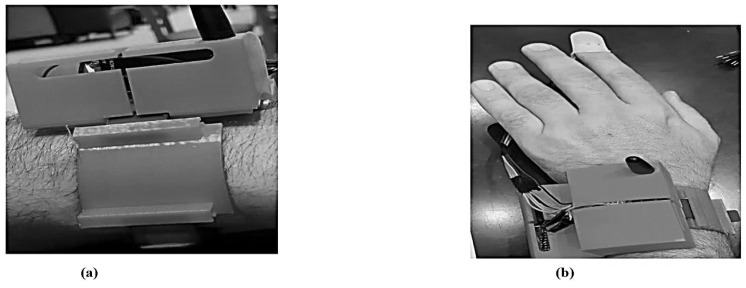
(**a**,**b**) Three-dimensional model of a wearable gadget.

Figure 11 shows the details of the STL file used only for 3D printing, along with the proper structure and dimensions of the design. The 3D model was made in SketchUp, then converted to an STL file before printing. Figure 11 displays the 3D model’s extra shape, dimensions, and data. The layers included for automated health data collection and patient satisfaction are shown in Figure 12 below.

The 3D design is affordable, lightweight, rigidly enclosed, and easy to use, and has a long lifespan. Figure 12 illustrates the levels of the sensing devices used to reduce stress on humans; the mechanism displays the results. The monitoring devices for sensing, regulating, and controlling COVID-19 are depicted in Figure 12. After being evaluated in real time, the design prototype is currently being utilized as an application hardware interaction for several test case situations with healthy and possibly infected patients in a hospital.

### 4.9. Comparison with Other ML Models

In the context of classification tasks, TP, FP, and FN are performance metrics that are used to assess the accuracy and effectiveness of a predictive model. The following provides an explanation of each term:

#### 4.9.1. True Positives (TPs)

True Positives refer to the number of instances that are correctly classified as positive by the model. In other words, these are the cases where the model predicted a positive outcome, and the actual ground truth is also positive. TP represents the successful identification of positive instances.

#### 4.9.2. False Positives (FPs)

False Positives represent the number of instances that are incorrectly classified as positive by the model. These are cases where the model predicted a positive outcome, but the actual ground truth is negative. FP represents instances that were mistakenly identified as positive when they should have been negative.

#### 4.9.3. False Negatives (FN)

False Negatives represent the number of instances that are incorrectly classified as negative by the model. These are cases where the model predicted a negative outcome, but the actual ground truth is positive. FN represents instances that were mistakenly identified as negative when they should have been positive.

These metrics are typically used together to evaluate the performance of a binary classification model. TP and FP are associated with the positive class, while FN is associated with the negative class. The combination of these metrics helps to assess the model’s ability to accurately identify positive instances (TP), the presence of false alarms (FP), and the instances that were missed or incorrectly classified as negative (FN).

These metrics are often used to calculate other evaluation measures such as precision, recall, and F1-score, which provide more comprehensive insights into the model’s performance.

The classifiers proposed in this study outperform the previously published findings. Additionally, some limitations depend on a specific strategy called feature selection. An example is given to demonstrate the reliance on this effort, as shown in the very accurate results in Table 6. Additionally, there is a negative effect when the datasets have a higher number of missing values. This model must handle the issues when the missing values are somewhat notable. Even if the training dataset is significantly expanded in the proposed approach, a larger dataset is still required to more specifically construct the model.

The performance measures of TP, FP, FN, precision, recall, and accuracy are all compared in Table 4. The average prediction results from the proposed model are precision of 96.8%, recall of 97.75%, and accuracy of 97.7% (see Figure 2, Figure 3 and Figure 4). Here, a sequential iteration of 100 epochs is taken into consideration, and the table above contains samples of 12 iterations. For 12 iterations, the average TP is 630, the FP is 312.5, and the FN is 23. In Iteration 1, the model achieved 600 true positives, indicating that it correctly identified 600 positive instances. There were seven false positives, meaning that seven instances were incorrectly classified as positive. Additionally, there were 18 false negatives, indicating that the model failed to identify 18 positive instances. The precision of this iteration was 99%, meaning that the model had a high proportion of correctly identified positive instances among all instances predicted as positive. The recall was also 99%, indicating that the model effectively captured a large proportion of actual positive instances. The accuracy for this iteration was 97.5%, reflecting the overall correctness of the model’s predictions.

In Iteration 2, the model improved its performance with 610 true positives and a reduced number of false positives (5). However, there were 30 false negatives, indicating some missed positive instances. The precision increased to 99.5%, indicating a higher proportion of correctly identified positive instances among all predicted positives. The recall decreased slightly to 97%, suggesting that the model missed a small portion of actual positive instances. The accuracy remained at 97%.

In Iteration 3, the model achieved 640 true positives, but the number of false positives increased to 19. There were 48 false negatives, indicating a larger number of missed positive instances compared to the previous iteration. The precision remained at 99%, indicating a high proportion of correctly identified positive instances among predicted positives. However, the recall decreased to 95%, indicating that the model failed to capture some actual positive instances. The accuracy dropped to 93%, reflecting the overall correctness of the model’s predictions. The analysis of each subsequent iteration followed a similar pattern, with varying numbers of true positives, false positives, and false negatives. The precision, recall, and accuracy metrics fluctuated across iterations, reflecting the model’s performance. The “Overall” row provides the average values across all iterations, showing that the model achieved an average precision of 96.8%, an average recall of 97.75%, and an average accuracy of 97.7%.

By evaluating these metrics for each iteration, we can assess the model’s performance in terms of correctly identifying positive instances, avoiding false positives and false negatives, and achieving overall accuracy in its predictions. Comparing the model performance to that of different existing methodologies, the proposed model is better. These findings show that the suggested model performs well in remote prediction and creates a better trade-off. This is similar to how better feature selection approaches enhance prediction quality.

Table 7 provides a comparative analysis of different algorithms, including the proposed algorithm. It considers various factors such as FP rate, TP rate, detection rate, precision, and F-measure. The results clearly demonstrate that the proposed ACAM algorithm outperforms the existing methods, showcasing its superiority. Furthermore, the table illustrates a comparison between the detection effectiveness of the suggested CNN-UUGRU technique and the current NN technique in terms of attack detection rate. The analysis focuses on different techniques, namely, 1NN, 2NN, 3NN, and 4NN. The findings reveal a substantial improvement in the detection rate of the proposed approach, with a significant increase of 35% compared to the existing techniques.

## 5. Conclusions

This research work proposed the innovative CNN-UUGRU integrated deep learning model for categorizing complicated human activities. In this investigation, raw sensor data from the WISDM dataset were employed. The 3D prototype model for an automated healthcare system, with the aim of decreasing stress and improving communications, comprises a detector, online API layer, and smartphone front-end layer. Measurements of the temperature, pulse, oxygen level, and sneeze frequency are performed with the use of wearable sensor layers. Each layer has a unique functionality. To relieve the family’s tension, the patient’s GPS location information is also sent to the appropriate health experts in real time. The application’s programmable logic layer is in charge of storing, gathering, and analyzing the data needed to manage the individual’s social life during the epidemic. Deploying wearable technology has been proposed as a model for screening airport visitors at arrival and departure. This work has undergone rigorous analysis to deliver the gadget with the highest performance possible by evaluating the existing domains. The novel elements of this design are the measurement of health disorders, following and closely monitoring of the patient throughout isolation, keeping data to foresee scenarios, swift alerting of authorities for efficient evaluation, and use of Android to educate family members about the patient’s status. The proposed model gives average prediction outcomes of 96.8% precision, 97.75% recall, and 97.7% accuracy. Furthermore, a successive iteration for 100 epochs is considered where the samples of 12 iterations are provided. IoT devices are targets for scammers, hackers, and other unethical individuals who are captivated by the vast amount of information disseminated across these devices. These data might be extremely harmful to anyone involved if they are accessed inappropriately. An automation algorithm that considers IoT security is therefore required for the future.

### Limitation and Future Work

The issue of fraudulent activities in healthcare systems based on the Internet of Things (IoT) is also addressed. Smart medical systems offer significant benefits to patients with chronic conditions compared to traditional healthcare services. However, one challenge is that remote areas often lack access to the necessary electricity required to power IoT devices. Additionally, the need for a fast and reliable network connection poses difficulties for real-time monitoring, especially in low-powered devices and remote locations. These limitations are not solely dependent on technological advancements and can be overcome with suitable solutions.

However, it is important to acknowledge that IoT devices are attractive targets for scammers, hackers, and other unethical individuals due to the vast amount of sensitive information transmitted through these devices. If these data are accessed inappropriately, there may by severe consequences for everyone involved. Therefore, there is a need for advanced IoT security measures, including automated algorithms, to address these concerns in the future.

## Figures and Tables

**Table 2:** Priority of physiological signals.

Signals of Physiological	Rate of Data	Level of Priority	Latency
EKG	Higher level	1	Minimum
Blood flow, heart rate, oxygen saturation	Lower level	2	Minimum
Blood pressure, body temperature, rate of respiration	Lower Level	3	Maximum
Never potentials	Higher Level	4	Maximum

**Table 3:** Hyper-parameters for CNN-UGRU.

Factors	Ranges
Conv filters 1	32
Conv filters 2	128
Dropouts 1	0.09
UGRU units 1	64
UGRU units 2	64
Dropout 2	0.01
Epochs	25
Size of batch	64

**Table 5:** Hyper-parameters for Deep_Convolutional-LSTM.

Factors	Ranges
Filters	[96, 44, 43, 52, 79, 14, 18]
Rate of learning	0.0008345
LSTM-Dims	[79, 57, 47]
Rate of regularization	0.006087

**Table 6:** Prediction outcomes.

Iterations	TP	FP	FN	Precision	Recall	Accuracy
1	600	7	18	99	99	97.5
2	610	5	30	99.5	97	97
3	640	19	48	99	95	93
4	655	35	17	96	99	97
5	590	43	9	94	99	98
6	660	25	9	99	99.5	99
7	660	30	20	98	99	97
8	660	77	11	97	99.5	99
9	640	63	12	92	99	99
10	580	14	31	94	97	99
11	670	25	55	98	95	99
12	600	36	40	97	95	99
Overall	630	31.5	23	96.8	97.75	97.7

**Table 7:** Proposed CNN-UUGRU and existing method comparison.

Techniques	Rate of TP	Rate of FP	Precision	Rate of Detection	F-Measure
1NN	32	31	27	18	81
2NN	35	39	18	21	84
3NN	26	39	14.3	15	82
4NN	42	24.5	24.7	19	71.8
CNN-UUGRU	98	6	96.5	24	97

## Data Availability

The data presented in this study are available on request from the corresponding author.

## References

[1] Spigulis J. (2017). Multispectral, Fluorescent and Photoplethysmographic Imaging for Remote Skin Assessment. Sensors.

[2] Sandryhaila A., Moura J.M. (2014). Big Data Analysis with Signal Processing on Graphs: Representation and processing of massive data sets with irregular structure. IEEE Signal Process. Mag..

[3] Qiu J., Wu Q., Ding G., Xu Y., Feng S. (2016). A survey of machine learning for big data processing. EURASIP J. Adv. Signal Process..

[4] Al Bassam N., Hussain S.A., Al Qaraghuli A., Khan J., Sumesh E., Lavanya V. (2021). IoT based wearable device to monitor the signs of quarantined remote patients of COVID-19. Informatics Med. Unlocked.

[5] Gani M.O. (2017). A Novel Approach to Complex Human Activity Recognition. Ph.D. Thesis.

[6] Vrigkas M., Nikou C., Kakadiaris I.A. (2015). A Review of Human Activity Recognition Methods. Front. Robot. AI.

[7] Chen K., Zhang D., Yao L., Guo B., Yu Z., Liu Y. (2020). Deep learning for sensor- based human activity recognition: Overview, challenges and opportunities. ACM Comput. Surv. CSUR.

[8] Preethi P., Asokan R. (2021). Modelling LSUTE: PKE Schemes for Safeguarding Electronic Healthcare Records Over Cloud Communication Environment. Wirel. Pers. Commun..

[9] Seshadri D.R., Li R.T., Voos J.E., Rowbottom J.R., Alfes C.M., Zorman C.A., Drummond C.K. (2019). Wearable sensors for monitoring the physiological and biochemical profile of the athlete. npj Digit. Med..

[10] Wang H., Zhao J., Li J., Tian L., Tu P., Cao T., An Y., Wang K., Li S. (2020). Wearable Sensor-Based Human Activity Recognition Using Hybrid Deep Learning Techniques. Secur. Commun. Netw..

[11] Huifeng W., Kadry S.N., Raj E.D. (2020). Continuous health monitoring of sportsperson using IoT devices based wearable technology. Comput. Commun..

[12] Jaber M.M., Alameri T., Ali M.H., Alsyouf A., Al-Bsheish M., Aldhmadi B.K., Ali S.Y., Abd S.K., Ali S.M., Albaker W. (2022). Remotely monitoring COVID-19 patient health condition using metaheuristics convolute networks from IoT-based wearable device health data. Sensors.

[13] Al Mamun M.A., Yuce M.R. (2019). Sensors and systems for wearable environmental monitoring toward IoT-enabled applications: A review. IEEE Sens. J..

[14] Reza Farsh S.M., Yaghoobi M. (2011). Fuzzy Logic Expert Systems in Hos-pital: A Foundation View. J. Appl. Sci..

[15] Preethi P., Asokan R., Thillaiarasu N., Saravanan T. (2021). An effective digit recognition model using enhanced convolutional neural network based chaotic grey wolf optimization. J. Intell. Fuzzy Syst..

[16] Muminov A., Mukhiddinov M., Cho J. (2022). Enhanced Classification of Dog Activities with Quaternion-Based Fusion Approach on High-Dimensional Raw Data from Wearable Sensors. Sensors.

[17] Hussain S.J., Khan S., Hasan R., Hussain S.A. (2020). Design and Implementation of Animal Activity Monitoring System Using TI Sensor Tag. Cognitive Informatics and Soft Computing.

[18] Preethi P., Asokan R. (2019). An attempt to design improved and fool proof safe distribution of personal healthcare records for cloud computing. Mob. Netw. Appl..

[19] Kulurkar P., Dixit C.K., Bharathi V., Monikavishnuvarthini A., Dhakne A., Preethi P. (2023). AI based elderly fall prediction system using wearable sensors: A smart home-care technology with IOT. Meas. Sensors.

[20] Chen H., Wang X., Ge B., Zhang T., Zhu Z. (2023). A Multi-Strategy Improved Sparrow Search Algorithm for Coverage Optimization in a WSN. Sensors.

